# Bromocriptine mesylate: FDA-approved novel treatment for type-2 diabetes

**DOI:** 10.4103/0253-7613.56070

**Published:** 2009-08

**Authors:** Rajiv Mahajan

**Affiliations:** Department of Pharmacology, AIMSR, Bhatinda, India. E-mail: drrajivmahajan01@yahoo.co.in

The food and drug administration (FDA), on May 5, approved bromocriptine mesylate 0.8-mg tablets as an adjunct to diet and exercise to improve glycemic control in adults with type-2 diabetes mellitus. This drug has been developed by Vero Science Inc, under the trade name *Cycloset*. The product is a quick-release formulation of bromocriptine, a centrally-acting dopamine D_2_ receptor agonist.[1] Bromocriptine is otherwise approved for the treatment of hyperprolactinemia-associated dysfunctions, acromegaly and Parkinson disease.[2] The idea of using bromocriptine for the treatment of type-2 diabetes came while studying the metabolism of migrating birds; that they develop seasonal insulin resistance and dopamine plays a role in it.[3] The results of phase 3b trial, conducted in response to a letter from FDA requesting additional data on bromocriptine cardiovascular safety, were declared in the second quarter of 2007[2] and finally approved recently.

**Figure 1:**
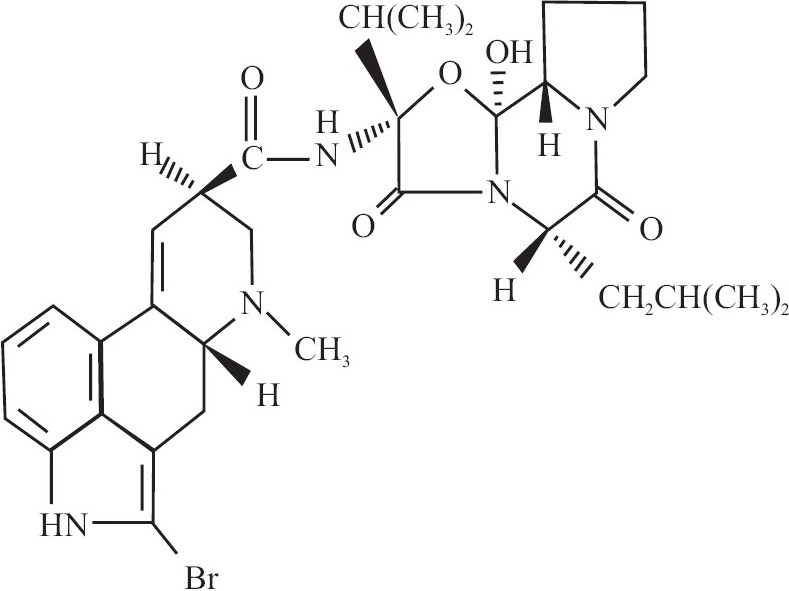
Structure of bromocriptine

Preclinical data suggest that decreased hypothalamic dopaminergic tone may be involved in the pathogenesis of insulin resistance. The normal circadian cycle that results in a leaner body in the summer and heavier body in winter is disrupted in humans because of abundant caloric intake year-round resulting in the absence of a lean phase. Stimulation of the hypothalamus promotes the release of several hormones that respond to the traditional shift in caloric intake and storage. Quick-release bromocriptine (D_2_ agonist), given once in the morning, stimulates the hypothalamus to release cortisol, growth hormone, and prolactin, allowing a reset of the circadian clock permanently stuck in a winter rhythm.[2] This means there occurs resetting of abnormally elevated hypothalamic drive for increased plasma glucose, triglyceride and free fatty acid levels in fasting and postprandial states in insulin-resistant patients[1][Figure 1].

Bromocriptine mesylate is indicated as an adjunct to diet and exercise to improve glycemic control in adults with type-2 diabetes mellitus. It may be used as monotherapy or as adjunctive therapy to metformin/sulfonylurea and single or dual oral hypoglycemic agent therapies. It should not be used to treat type-1 diabetes or diabetic ketoacidosis. There is limited efficacy data in combination with thiazolidinediones and efficacy has not been confirmed in combination with insulin.[4]

Bromocriptine is the first drug for treatment of diabetes to be approved under the FDA's new guidelines which require clinical trials to demonstrate no increased cardiovascular risk.[1] In a 52-week, double-blind, placebo-controlled safety trial (n = 3070), treatment with quick-release formulation of bromocriptine reduces the incidence of diabetic cardiovascular complications in patients with type-2 diabetes and improves glycemic control in those patients who did not achieve HbA_1c_ of less than 7.5% with metformin plus a sulfonylurea. The incidence of the composite cardiovascular end point of myocardial infarction (MI), stroke, coronary revascularization, and hospitalization for congestive heart failure (CHF) or unstable angina was 1.5% among patients receiving bromocriptine and three percent among patients receiving placebo.[2]

The recommended starting dose of bromocriptine is 0.8 mg daily and increased in 0.8 mg increments weekly until the target range (1.6 - 4.8 mg) or till maximal tolerance is reached. Doses should be administered once daily within two hours of waking in the morning and with food to reduce the risk for gastrointestinal adverse effects such as nausea.[1] Studies suggest that one morning dose helped lower the usual post-meal blood sugar rise at breakfast, lunch and dinner. Over six months, 35% of drug users reached recommended average blood sugar levels compared with 10 per cent of diabetics given placebo.[3]

The drug is contraindicated in patients with known hypersensitivity to bromocriptine or ergot-related drugs. It is also contraindicated in patients with syncopal migraine. Bromocriptine increases the likelihood of a hypotensive episode among patients with syncopal migraine. Loss of consciousness during migraine may reflect dopamine receptor hypersensitivity. Bromocriptine is a dopamine receptor agonist, and may, therefore, potentiate the risk for syncope in these patients.[4] It is also contraindicated in nursing women. Bromocriptine may inhibit lactation. There are postmarketing reports of stroke in these patients although causality has not been proven.[3]

The most common adverse events associated with bromocriptine mesylate are nausea, fatigue, dizziness, vomiting and headache. Clinical trial data reveal that bromocriptine mesylate at doses up to 4.8 mg per day was not associated with a different rate of all-cause adverse events compared with placebo. The incidence of hypoglycemia was 6.9% among bromocriptine mesylate-treated patients compared with 5.3% of patients receiving placebo.[5]

The FDA warns that bromocriptine can cause orthostatic hypotension and syncope, particularly on initiation of therapy and dose escalation. Caution is advised when treating patients who are receiving antihypertensive therapy; orthostatic vital signs should be evaluated at baseline and periodically thereafter.[1]

Several oral hypoglycemic agents are already available for type-2 diabetic patients to normalize plasma glucose concentration. However, they have not had complete and sustained success. Some benefits of quick release bromocriptine mesylate therapy include:

- Resetting of circadian rhythm, a novel mechanism of action very different from currently used antidiabetic drugs
- Use with other antidiabetic agents
- Ease of a single morning daily dose
- Lower incidence of MI, stroke, and vascular events unlike other antidiabetic agents.

Due to these beneficial effects bromocriptine mesylate may prove to be a revolution in the field of treatment of type-2 diabetes.
